# Goal-Directed Use of Prothrombin Complex Concentrates in Liver Transplantation: Is a Plasma-Free Procedure Feasible?

**DOI:** 10.3390/hematolrep16030044

**Published:** 2024-07-08

**Authors:** Giovanni Punzo, Valeria Di Franco, Paola Aceto

**Affiliations:** 1 Department of Anesthesiology and Intensive Care Medicine, Fondazione Policlinico Universitario “A. Gemelli”, IRCCS, Università Cattolica del Sacro Cuore, Largo A. Gemelli 8, 00168 Rome, Italy; difrancovaleria@gmail.com (V.D.F.); paola.aceto@policlinicogemelli.it (P.A.); 2 Department of Basic Biotechnological Science, Intensive and Peri-Operative Clinics, Università Cattolica del Sacro Cuore, Largo Francesco Vito 1, 00168 Rome, Italy

**Keywords:** prothrombin complex concentrates, fresh frozen plasma, liver transplantation, coagulopathy, coagulation management

## Abstract

**Background:** Fresh frozen plasma (FFP) transfusions have been the mainstay of hemostatic intervention for the treatment of bleeding and coagulation abnormalities arising during liver transplantation (LT) for decades. However, numerous clinical studies showed that FFP has many side effects, including the risk of pathogen transmission, transfusion-associated circulatory overload (TACO), transfusion-related immunomodulation (TRIM), and transfusion-related acute lung injury (TRALI). These adverse events are particularly challenging in patients undergoing LT, who often suffer from severe portal hypertension, poor renal function and coexisting cardiac disease.The aims of this review are to summarize the pharmacological properties of currently available PCCs, to represent the theoretical benefits and the possible risks related to the use of these drugs in patients undergoing LT, and, finally, to review the current literature on the topic in order to highlight the evidence that currently supports PCC use in LT patients. **Methods:** The current literature on the topic was reviewed in order to highlight the evidence that currently supports PCC use in LT patients. **Results:** Prothrombin complex concentrates (PCCs) may offer several advantages when compared to FFP. Indeed, PCCs have been shown to reduce the risk of TACO, which during liver transplantation may deteriorate portal hypertension, increase intraoperative bleeding, and possibly reduce survival rates. One of the major concerns for PCC use is thrombogenicity. However, currently available PCCs are much safer as they contain inactivated forms of the vitamin K-dependent coagulation factors and protein C, protein S, antithrombin and/or heparin. Nowadays, the use of PCCs to correct coagulation abnormalities that occur during LT is an increasingly widespread practice. However, it is not yet clear what level of evidence supports this practice, and what the risks associated with it are. **Conclusions:** Administration of PCC in LT patients to correct haemostatic abnormalities seems to be well-tolerated, but the relationship between PCC use and thromboembolic events in the postoperative period remains unclear. Adequately powered, methodologically sound trials are urgently required for more definitive conclusions about the efficacy and safety of PCCs in a broad phenotype of LT recipients.

## 1. Introduction

Prothrombin complex concentrates (PCCs) are frequently used to correct coagulation abnormalities in patients with acute or chronic liver disease undergoing liver transplantation (LT) [1,2,3,4,5,6,7,8,9]. The main reason for this practice is the multiple advantages that PCC use might have in LT patients, particularly when compared to fresh frozen plasma (FFP) or other pro-coagulant drugs. To give an example, intraoperative PCC administration allows for a rapid increase in plasma levels of vitamin K-dependent clotting factors—and consequently, rapid correction of the international normalized ratio (INR)—without having to administer a large volume of fluids. Therefore, correction of hemostatic defects by infusion of PCCs may potentially reduce the risk of intraoperative transfusion-associated circulatory overload (TACO). This complication is extremely dangerous in LT recipients as it may worsen portal hypertension, increase intraoperative bleeding, and consequently reduce survival [1,10,11,12].

However, PCC use in LT patients may also increase the risk of thrombotic and thromboembolic events [3,13,14], which has not been systematically examined by previous investigations.

The aims of this study are to summarize the pharmacological properties of currently available PPCs, to represent the theoretical benefits and the possible risks related to the use of these drugs in patients undergoing LT, and, finally, to review the current literature on the topic in order to learn more about the benefit/risk ratio of the increasingly widespread practice of correcting coagulation abnormalities that occur during LT by administering PCC.

## 2. PCC Characteristics

PCCs comprise a heterogeneous group of plasma-derived products containing partly purified vitamin k-dependent clotting factors. Three different types of these products are available for clinical use: three-factor PCC (3F-PCC), four-factor PCC (4F-PCC), and activated PCC. The 3F-PCCs contain vitamin k-dependent clotting factors II, IX, and X. The 4F-PCCs, compared to 3F-PCC, also contain therapeutic amounts of factor VII. Activated PCC (Feiba^®^), compared to 3F-PCC and 4F-PCC, comprises the activated factor VII (VIIa) in addition to the pro-enzymes prothrombin (factor II), factor IX, and factor X. All products may or may not contain small quantities of anticoagulants like protein C, protein S, and protein Z as well as small amounts of antithrombin (AT III) and heparin to mitigate thrombogenicity and prevent the activation of vitamin k-dependent clotting factors (Table 1).

In general, 3F-PCCs should be less effective than those products containing clotting factors for the reversal of anticoagulation by vitamin K-antagonists, probably due to the lack of factor VII. PCCs are usually available as 500- or 1000-unit (IU) vials; the dosage refers to the factor IX content. In contrast, the potency of activated PCCs is expressed in arbitrary units defined as the amount able to reduce the clotting time of factor VIII inhibitor to 50% of the normal amount in the reference plasma. According to the European Pharmacopoeia guidelines [15], a PCC should have a factor IX potency of at least 20 IU/mL, and the relative potencies of clotting factors II and X should be close to factor IX but not exceed it by more than 20%. All PCCs present as lyophilized powder to be reconstituted with normal saline solution and then injected intravenously. 

The 3F-PCCs have been approved in several countries including the United States for hemophilia B treatment. Instead, the 4F-PCCs have been approved by the Food and Drug Administration for any vitamin K-dependent factor deficiencies and the Haemostasis and Transfusion Scientific Subcommittee of the European Association of Cardiothoracic Anaesthesiology recommends their use for cardiac and non-cardiac surgical patients with massive bleeding and coagulopathy (characterized by a clotting time prolongation of a tissue-factor-activated viscoelastic point-of-care test: EXTEM or EXtest) [16]. In these patients, the recommended initial dose is 25 IU/kg, to be administered after a fibrinogen deficit has been ruled out (via normal FIBTEM/FIBtest clot firmness amplitude) [17]. In cases of patients at high risk of thromboembolic complications (e.g., cardiac surgery or liver transplantation) the administration of an initial half-dose bolus (12.5 IU/kg) should be considered. A second bolus may be indicated if coagulopathy and microvascular bleeding persists and other reasons for bleeding are largely ruled out [17].

Activated PCC is indicated for the treatment and prophylaxis of bleeding in patients with inhibitors of factors VIII or IX, regardless of the diagnosis of hemophilia A and B. The efficacy of PCC has not been established yet in pregnant or nursing women or pediatric populations.

## 3. Rationale for PCC Use in LT Patients

End-stage liver disease (ESLD) leads to a complex pathophysiologic state of “rebalanced hemostasis” in which the coagulation system is adversely affected by low levels of pro- and antithrombotic factors at the same time. Deficiency in coagulation factors such as factor V, VII, IX, X, XII, prothrombin (II), and fibrinogen (I) would suggest a bleeding tendency, whereas cirrhotic patients may still have normal thrombin-generating capacity due to the decreased production of the natural anticoagulants protein C, co-factor S, antithrombin III, and increased levels of endothelium-derived factor VIII. Furthermore, despite a reduction in platelet count or function (due to hypersplenism secondary to portal hypertension, reduced synthesis of thrombopoietin by the liver, bone marrow suppression, increased endothelial production of nitric oxide and prostacyclin, and uraemia), platelet activity is often increased in liver disease as von Willebrand Factor (vWF), a platelet adhesive protein produced in endothelial cells, is often elevated and ADAMTS-13, a vWF-cleaving protease produced by the liver, is often decreased. 

Although the net results of these major changes in the hemostatic system depend on multiple factors, such as the rate at which liver disease develops or the underlying cause of it, diffuse bleeding due to coagulopathy is rarely observed in stable patients with liver disease, signaling that a new hemostatic balance is usually reached. However, this new hemostatic rebalanced state is also precarious, and perturbations associated with the perioperative period during LT can easily decompensate the delicate balance in the hemostatic system of these patients towards either bleeding or thrombotic tendency. 

In this context, FFP transfusions have been a mainstay of hemostatic intervention for the prevention and treatment of bleeding and coagulation disorders arising during LT for decades, often without clearly defined therapeutic goals. In fact, in the setting of LT, FFP has usually been used to lower the INR, but it is now well established that the INR is unable to fully evaluate the complex hemostatic status of patients with ESLD undergoing LT [18]. Moreover, FFP use has many limitations [10,11,19,20,21,22]. 

First of all, although the risk of pathogen transmission is low, the use of allogenic plasma is still associated with other complications, including TACO, transfusion-related acute lung injury (TRALI), and transfusion-related immunomodulation (TRIM) [10,21,22,23]. These complications are poorly tolerated by patients undergoing LT, especially those with severe portal hypertension, poor renal function, and coexisting cardiac disease (which is quite common in LT recipients) [10,11,21]. 

Another important consideration is that fluid overload by intraoperative transfusion of a large volume of FFP and other blood products may paradoxically worsen surgical bleeding by increasing central venous pressure and portal hypertension, especially in the pre-anhepatic phase of LT [2,12,19,24,25,26]. Furthermore, the transfusion of a large volume of FFP should be carefully avoided in the neo-hepatic phase of LT, as it may induce an excessive increase in central venous pressure and consequently compromise graft perfusion [2,10,19,24]. Therefore, the haemostatic effects of FFP transfusions might be counteracted by bleeding complications and other adverse effects due to circulatory overload.

**Figure 1:**
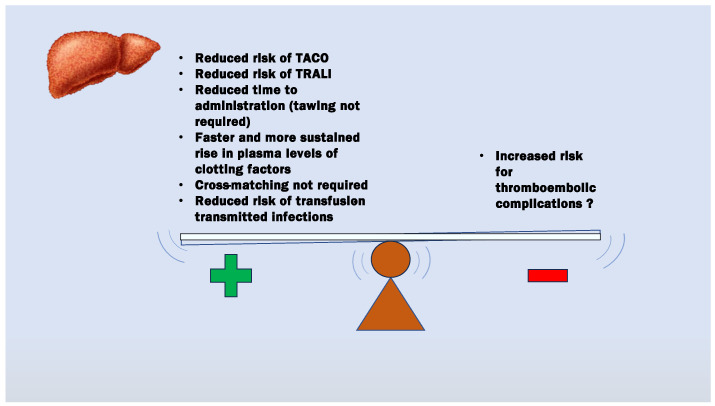
Potential advantages (+) and disadvantages (−) of PCC administration for the correction of coagulation abnormalities in LT patients when compared to FFP.

PCC and other factor concentrates (FC) such as fibrinogen concentrate (FibC) offer several advantages compared to FFP (Figure 1). The major advantage of PCC over FFP is the use of a smaller volume of product required to reverse anticoagulation. Indeed, in cases of severe bleeding, the commonly recommended dose of FFP for reversing INR to the target value of 1.5 is, for most patients, approximately 10–30 mL/kg [2,10,12]. At these doses, a 70 kg patient might receive a volume of 700–2100 mL of FFP, which may significantly increase the risk of circulatory overload. As the administration of 1 IU/kg of PCC usually results in a 0.6–1% increase of the activity of the corresponding coagulation factors [2], the same effect of the abovementioned large volume of plasma on INR can be usually achieved by a dose not exceeding 10–30 IU/kg of PCC. This dose can be delivered in injection volumes equal to no more than 1–2 mL/kg, which are usually well tolerated by the surgical patient. 

Moreover, PCCs are unlikely to provoke TRALI, which is the second most common cause of death among LT patients, only surpassed by sepsis, with mortality rates over 25%. [22,24] In this regard, platelet and FFP transfusions have been previously reported as major risk factors for TRALI [22,23]. In one widely accepted model for mechanisms of TRALI, alloantibodies in the transfused donor blood products cause the activation of the recipient’s neutrophils, leading to an inflammatory process causing lung injury [23,24]. As PCCs are free of leukocytes and alloantibodies, TRALI has never been documented as a safety concern with PCC use in the perioperative setting.

Another major advantage of PCC use in surgical patients, including those undergoing LT, is the significant advantage in terms of time to administration, compared to FFP. Indeed, unlike FFP, PCCs are usually stored at room temperature and do not require thawing. Whereas the infusion of 2000 mL of FFP usually requires 60–90 min (40 min infusion plus thawing time), the infusion of 2000 IU PCCs can be accomplished within max 10–20 min. Thus, as PCCs can be quickly administered to the patient, they might allow for a more rapid correction of the coagulation defects that frequently occur during LT. Moreover, PCCs are supposed to allow a markedly faster and more sustained rise in plasma levels of clotting factors compared to FFP [27,28,29,30]. Pharmacological data demonstrate the rapid normalization of vitamin K-dependent factors within 30 min using PCCs in warfarin-treated patients, while plasma transfusion requires at least 3 h [30,31]. Prothrombin (factor II) and factor X are usually returned to 80–100% activity in patients receiving a single dose of PPCs, and these levels are maintained for the next 24 h after PCC administration [31]. Such effects, in terms of potency and duration of action, are usually not observed in patients receiving equivalent doses of FFP. 

PCCs do not require cross-matching, can be easily reconstituted from the lyophilized form, and have a transfusion-transmitted infection risk of almost zero. The available FFP is associated with a low risk of transfusion-transmitted infection because it is generally not virally inactivated. PCC formulations undergo at least one procedure of viral reduction, and most of them two steps. No case of transfusion-transmitted disease has been reported with PPC use since the mid-1990s.

One of the major concerns for PCC use in surgical patients, including those undergoing LT, is thrombogenicity and potential for thromboembolic complications [3,13,14]. These complications may be increased by the patients’ underlying conditions, high or repeated dosing, coadministration of other hemostatic therapies, the presence of activated procoagulant factors, and poorly balanced procoagulant and anticoagulant proteins in PCCs. However, currently available PCCs are much safer than older preparations because of improved manufacturing procedures used to maintain inactivated forms of PCC factors and the inclusion of protein C, protein S, ATIII and/or heparin. In a recent meta-analysis of 17 studies including a sample of 2745 patients undergoing major surgery, with 156 patients undergoing LT, PCC use did not result in an increased incidence of thromboembolic events [32]. However, this finding was derived from a small number of retrospective studies and therefore should be interpreted with caution. More recently, in a single-center, retrospective study on 939 patients undergoing LT, PCC and FibC administration were independently associated with early onset of hepatic artery, portal vein, and/or inferior vena cava thrombosis within the first 30 days after surgery [3].

Since PT and aPTT proved to be poor indicators of clot formations in patients with chronic liver disease and are unrelated to bleeding risk in these patients, international recommendations and guidelines (e.g., LICAGE 2019) recommend not using PT/INR to guide PCC administration in patients with chronic liver disease and coagulopathy in liver transplantation [18]. Tissue-factor-activated, factor VII-dependent and heparin-insensitive point-of-care tests (EXTEM or EXtest) should therefore be used to monitor coagulation and guide PCC administration in these patients [18]. 

## 4. Evidence of Benefits from Coagulation Factor Concentrate Therapy in LT

In a pioneering study published in 1975, Gazzard and colleagues compared the usefulness of PCC and FFP in the treatment of haemostatic defects in patients with chronic liver disease [33]. In this study, 30 patients with a prolonged prothrombin time requiring liver biopsy for diagnostic purposes were treated with 900 mL FFP or 2000 IU of Prothromplex (Serological Products Ltd, Dunton Green, Kent, UK.) containing factors II, VII, and X, and a small quantity (less than 85 units) of factor VII. The increase in the levels of coagulation factors was more pronounced in the group receiving Prothromplex, and this drug was found to be more effective than PCC in correcting prothrombin time. There was no clinical evidence of bleeding in any of the patients of the two groups, and no laboratory evidence of intravascular coagulation was found in the patients treated with FC.

One year later, these results were confirmed by Mannucci and colleagues [34], who compared the effect on abnormal coagulation tests of FPP infusion, PCC or a combination of these drugs in 30 patients with ESLD undergoing liver biopsy. In this study, treatment with 12 mL FFP/Kg led to an improvement but, in most cases, not to normalization of pathological coagulation tests, whereas substitution with PCC (Prothromplex, 25 IU/kg) resulted in a complete correction of the abnormal values of the coagulation tests except the activated partial thromboplastin time. There was no clinical or laboratory evidence of thrombotic complications in patients treated with PCC, and no bleeding episodes were observed.

The first reported use of PCC in patients undergoing LT was by Scherer and colleagues [35] in 1994. Fifty one adult patients with acute or chronic ESLD scheduled for LT were prospectively studied to investigate the pharmacokinetics of FC, and fourteen patients in this study received PCC. The main finding in this study was that a relatively higher dose of 1.6 IU PCC/kg has to be given in the presence of ESLD to increase the Quick percentage value by about 1%, versus 0.5–1 IU PCC/kg in patients without liver disease. PCC administered to all patients in this study was PPSB (Beriplex, Behring, Marburg, Germany), a plasma-derived, pasteurized and nanometer-filtered 4F-PCC containing large amounts of protein C (150–450 IU for vial). AT-III concentrate was given to all patients to increase the endogenous activity to 70–80%. No data on thromboembolic events were presented in this study.

More recently, in 2014, Kirchner and colleagues [4] published a large case series on the use of coagulation FC (PCC and FibC) in patients undergoing LT. Coagulation management in the FC group (n = 156 patients) was guided by a rotational thromboelastography (ROTEM)-based algorithm, and FC was used as a combined first-line therapy. According to the point-of-care algorithm used in this study, PCC administration—at a dose of 25 IU/kg—was triggered by an EXTEM clotting time of more than 80 s, and FibC administration was triggered by a maximum clot firmness (MCF) in EXTEM and FIBTEM analyses of less than 35 and 6 mm, respectively. FibC administration continued in the postoperative period, guided by conventional laboratory tests to maintain the fibrinogen concentration at 1.5 to 2.0 g/L and to achieve an INR of 2.3 as a maximum. At the end of the study, the mean dose of FibC administered in the FC group was 6.3 ± 5.6 g, and for PCC, it was 4090 ± 3130 IU (Table 2). Despite a higher MELD (Model for End-Stage Liver Disease) score in the FC group (23 vs. 17), a significant reduction in the median number of red blood cells (RBCs) transfused in this group was observed (from 3 to 0 per patient) compared to the non-coagulation factor group. No statistically significant difference between thromboembolic and ischemic events was registered between FibC/PCC-treated and non-treated cohorts until postoperative day 14 in this study (Table 2).

As in the above-mentioned study, the hypothesis that intraoperative use of PCC in LT patients may reduce intraoperative allogenic blood product requirements was also tested by Colavecchia and colleagues in 2017 [5]. In this retrospective, single-center study, 39 consecutive LT patients who received PCC in the perioperative period were propensity-matched at a 1 to 2 ratio with 78 LT patients not exposed to PCC. According to the study protocol, patients exposed to FC were defined as those who received PCC and/or FibC administration from 24 h before surgery to 72 h after the procedure. In the FC group, PCC and FibC were administered not according to any standardized protocol, and providers could freely decide timing, dose, type of product (PCC or FibC), and the possible concomitant use of allogenic blood products based solely on their clinical judgment. At the end of the study, the median dose of PCC administered in the exposed group was 902 ± 589 IU, and 33 (84.6%) of the 39 patients who received PCC received concomitant FibC at a mean dose of 1.679 ± 1.107 g. The authors concluded that PCC and FibC use during LT did not reduce intraoperative allogenic RBC, FFP, cryoprecipitate, or platelet concentrate requirements and was not associated with worse clinical outcomes, such as intensive care unit length of stay or acute kidney injury. The incidence of thrombotic and/or thromboembolic events in the two study groups was not reported.

Two large retrospective studies, both including a propensity score-matching analysis to minimize selection bias between patients who were exposed or not exposed to PCCs, were conducted in 2018. In the study by Srivastava and colleagues [6], 60 patients undergoing LT who received PCC during surgery were matched at a 1:1 ratio with patients who did not receive PCC in order to investigate the safety and efficacy of PCC as a first-line treatment for coagulopathy. In all patients, hemostatic therapy was based on point-of-care test results, according to a thromboelastography (TEG)-guided intraoperative coagulation protocol implemented by the authors. In the intervention group, a prolongation of the TEG R value (>10 min) was treated with PCC given at a dose of 25 IU/Kg. After surgery, Doppler ultrasonography was performed twice daily for the first 7 days to screen for thrombotic complications. The authors found a significant decrease in RBC and FFP transfusion requirements during LT in patients who had received PCC. Additionally, no differences in the number of postoperative days in mechanical ventilation and thrombotic and/or thromboembolic complications were found. Interestingly, the incidence of hemorrhagic complications requiring re-exploration was higher in the non-PCC population. The authors did not report the mean dosage of PCC administered during surgery in the study population. 

In the study by Zamper and colleagues [7], 46 liver transplanted patients exposed to FC during surgery were propensity-matched with 89 LT patients not exposed to these drugs. Patients exposed to FC were managed according to a ROTEM algorithm implemented by the authors for the treatment of coagulopathy during LT, which has a similar structure compared to that of Kirchner [4]. FibC was administered to 16 patients in the intervention group at a median dose of 1.4 ± 2.4 g, whilst PCCs were administered only in 5 patients at a relatively low median dose of 195.6 ± 645.3 IU. The pharmacological characteristics of FC used in this study were not specified. The authors concluded that the ROTEM-guided coagulation management with FC significantly reduced the transfusion rate of any allogenic blood product, and no differences in the incidence of thromboembolic complications were found between the two cohorts of patients. However, the incidence of thromboembolic complications in the two study groups was not specified.

In 2019, Hartmann and colleagues [8] retrospectively analyzed 372 LT procedures performed over nearly 5 years. In their study, all patients were managed according to a ROTEM-guided coagulation management algorithm involving the use of FC, platelets, and fibrinolysis inhibitors without using FFP. The main aim of this study was to evaluate the influence of this plasma-free coagulation management algorithm on 30-day mortality in LT patients. PPC (KCentra, CSL Behring) was administered to 70 patients with doses ranging from 1000 to 7000 IU, and FibC was given to 187 patients using a dose ranging between 1 and 22 g. Multivariate analysis in this study demonstrated that platelet concentrates, blood loss, and MELD score (but not PPC and FibC) are independent predictors of mortality. This result supports the safety of FC. However, no data were reported in this study on the incidence of thromboembolic complications in the study population.

Finally, in a recent retrospective, single-center study, Dehne and colleagues [3] analyzed the safety of perioperative PCC and FibC administration in 939 patients who had undergone LT. PCC administration in this study was guided by viscoelastic tests (TEG or ROTEM), and patients in the FC group received 3350 ± 3500 IU PCC and 4.59 ± 4.6 g FibC on average. The specified primary endpoint was occurrence of early hepatic artery, portal vein and inferior vena cava thrombosis within the first 30 postoperative days, and it was more frequent in the FC group (11.3% vs. 6.6%, FC group vs. non-FC group, *p* = 0.017). The authors concluded that perioperative PCC and FibC administration is independently associated with thrombotic and thromboembolic complications in the perioperative period, and that the use of factor concentrates during liver transplantation must be critically reviewed and reconsidered. The type of FC used in this study was not specified.

## 5. Conclusions

Administration of PCC in patients undergoing LT to correct hemostatic abnormalities appears to be possible and well tolerated, and the use of four-factor PCC could have the potential to significantly reduce the perioperative use of allogenic blood products including FFP [9]. However, clinical evidence on the efficacy and safety of PPC administration in patients undergoing LT is low, and it has not been definitively ascertained whether or not this practice may increase the risk of thromboembolic complications in the perioperative period. There is no published randomized controlled trial on the topic, and the PROTON trial, which is the only such trial in Europe, has recently been stopped because it never included the planned number of patients [36]. High-quality studies are needed to ascertain the efficacy, safety, optimal dose and timing of PCC administration, and whether there is an optimal PCC composition for its use in the setting of LT. Future studies in this field should include screening for patients’ underlying conditions to assess the safety of PCCs in patients with a hypercoagulable condition such as hepatocellular carcinoma, Budd–Chiari syndrome, portal vein thrombosis, or primary sclerosing cholangitis. Moreover, studies comparing PCC with alternative therapies should be designed to have doses and therapeutic targets that are comparable across treatment arms. 

Finally, after ascertaining the current lack of robust evidence to support the use of PCC in patients undergoing LT, the levels of evidence for the alternative therapies, including FFP, should be carefully considered when choosing how to manage these patients. 

While awaiting robust evidence on the practice of both PCC and FFP administration, to provide the highest standard of care for our patients, it is important to tailor therapeutic choices on a case-by-case basis and not to confuse habits with evidence.

## Figures and Tables

**Table 1:** Composition of most commonly available PCCs, as reported by manufacturers.

	PCC (Manufacturer)	Factors (IU/mL)				Coagulation Proteins (IU/mL)			Other Antithrombotic Additions
		F. II	F. VII	F. IX	F. X	Protein S	Protein C	Protein Z	
3F-PCC	Bebulin^®^ (Baxter Healthcare Corporation, Westlake village, CA, USA)	24–37	<5	24–37	24–37	N/A	N/A	No	Small amounts of heparin (<0.15 IU per IU F.IX)
	Profilnine^®^ (Grifols Biologicals, Barcelona, Spain)	87	-	69	54	0	0	No	None
	UMAN Complex D.I. (Kedrion, Italy)	28	<0.1	28	21	5	9	Yes	Antithrombin, heparin
4F-PCC	Beriplex^®^/Confidex^®^/Kcentra^®^ (CSL Behring, Marburg, Germany)	20–48	10–25	20–31	22–60	17–19	22–31	Yes	Antithrombin, heparin, albumin
	Cofact^®^ (Sanquin, Amsterdam, The Netherlands)	30	13	23	26	21	4	Yes	Antithrombin
	Octaplex^®^ (Octapharma, Brussels, Belgium)	31	16	22	24	24	12	Yes	Heparin, low activated Factor VIIa
	Prothromplex T^®^ (Baxter Bioscence, Vienna, Austria)	12	11	8	11	8	4	Yes	Antithrombin, heparin

3F-PCCs: three-factor prothrombin complex concentrates; 4F-PCCs: four-factor prothrombin complex concentrates.

**Table 2:** Overview of studies on the use of coagulation factor concentrates (PCCs and FibCs) for the correction of coagulopathy in liver transplantation procedures.

Author (Year)	Study Type	Patients	PCC	3F-/4F-PCC	Mean Dose PCC	Coagulation Monitoring	Clinical Outcome	Incidence of Thrombotic and/or Thromboembolic Events (FC Group vs. the Non-FC Group)
Kirchner (2014) [4]	Retrospective study	266 (FC group n = 156)	Beriplex^®^	4F-PCC	4090 ± 3130 IU	ROTEM	Incidence of thrombotic, thromboembolic and ischemic events in the first 10 postoperative days	7.1% vs. 4.5% *p* = 0.31
Colavecchia (2017) [5]	Retrospective/ Propensity-matched	117 * (PCC group n = 39)	Kcentra^®^	4F-PCC	902 ± 589 IU	SCT	Reduction in allogenic blood product use	N/A
Srivastava (2018) [6]	Retrospective/Propensity-matched	120 * (PCC group n = 60)	N/A	N/A	N/A. 25 IU/kg given if TEG R > 10 min.	TEG	Reduction in allogenic blood product use	0.0% vs. 0.0%
Zamper (2018) [7]	Retrospective/Propensity-matched	135 * (PCC group n = 46)	N/A	N/A	195.6 ± 645.3 IU	ROTEM	Reduction in allogenic blood product use	N/A
Hartman (2019) [8]	Retrospective study	372 (PCC group n = 70)	Kcentra^®^	4F-PCC	N/A. Range 1000–7000 IU	ROTEM	Reduction in 30-day mortality in LT	N/A
Dehne (2022) [3]	Retrospective study	939 (FC group n = 576)	N/A	N/A	3350 ± 3500 IU	TEG or ROTEM	Occurrence of hepatic artery, portal vein or inferior vena cava thrombosis within the first 30 days after surgery	11.3% vs. 6.6% *p* = 0.017

PCC: prothrombin complex concentrate; FibC: fibrinogen concentrate; 3F-PCC: three-factor PCC; 4F-PCC: four-factor PCC; SCTs: standard coagulation tests; ROTEM: rotational thromboelastometry; TEG: thromboelastography; IU: units. Significance *p* < 0.05. * After propensity score-matching; N/A: Not applicable.

## Data Availability

Not applicable.
